# Fenoldopam for Renal Protection in Cardiac Surgery: Pharmacology, Clinical Applications, and Evolving Perspectives

**DOI:** 10.3390/jcm13195863

**Published:** 2024-10-01

**Authors:** Giuseppe Cuttone, Luigi La Via, Giovanni Misseri, Giulio Geraci, Massimiliano Sorbello, Federico Pappalardo

**Affiliations:** 1 Faculty of Medicine and Surgery, Kore University, 94100 Enna, Italy; giulio.geraci@unikore.it (G.G.); massimiliano.sorbello@unikore.it (M.S.); federico.pappalardo@unikore.it (F.P.); 2 Department of Anesthesia and Intensive Care 1, University Hospital Policlinico “G. Rodolico–San Marco”, 95123 Catania, Italy; luigilavia7@gmail.com; 3 Fondazione Istituto “G. Giglio” Cefalù, 90100 Cefalù, Italy; giovannimisseri1987@gmail.com; 4 Department of Anesthesia and Intensive Care, Giovanni Paolo II Hospital, 97100 Ragusa, Italy; 5 Policlinico Centro Cuore GB Morgagni, 95100 Catania, Italy

**Keywords:** acute kidney injury, dopamine-1 receptor agonist, ischemia–reperfusion injury, cardiopulmonary bypass, renal blood flow, pharmacological intervention, perioperative care

## Abstract

This comprehensive review examines the role of Fenoldopam, a selective dopamine-1 receptor agonist, in preventing and treating acute kidney injury (AKI) during cardiac surgery. AKI remains a significant complication in cardiac surgery, associated with increased morbidity, mortality, and healthcare costs. The review explores Fenoldopam’s pharmacological properties, mechanism of action, and clinical applications, synthesizing evidence from randomized controlled trials, meta-analyses, and observational studies. While some studies have shown promising results in improving renal function and reducing AKI incidence, others have failed to demonstrate significant benefits. The review discusses these conflicting findings, explores potential reasons for discrepancies, and identifies areas requiring further research. It also compares Fenoldopam to other renoprotective strategies, including dopamine, diuretics, and N-acetylcysteine. The safety profile of Fenoldopam, including common side effects and contraindications, is addressed. Current guidelines and recommendations for Fenoldopam use in cardiac surgery are presented, along with a cost-effectiveness analysis. The review concludes by outlining future research directions and potential new applications of Fenoldopam in cardiac surgery. By providing a thorough overview of the current state of knowledge, this review aims to facilitate informed decision-making for clinicians and researchers while highlighting areas for future investigation.

## 1. Introduction

Cardiac surgery, while life-saving, carries significant risks, including acute kidney injury (AKI). AKI incidence post-cardiac surgery varies from 8.9% to 39%, based on the definition and patient population [1,2]. This complication leads to increased morbidity, mortality, hospital stay duration, and healthcare expenses [3,4]. Fenoldopam, a selective dopamine-1 receptor agonist, has emerged as a potential renoprotective agent in cardiac surgery [5]. Initially approved for severe hypertension treatment, Fenoldopam has been studied for its capacity to enhance renal blood flow and urine output, potentially reducing AKI risk in high-risk patients [6,7]. Kidneys are especially susceptible during cardiac surgery due to factors such as hemodynamic alterations, ischemia–reperfusion injury, inflammation, and oxidative stress [8,9]. These insults may cause AKI, impacting not only short-term outcomes but also potentially leading to long-term consequences like chronic kidney disease [10]. Thus, strategies for renal function protection during and after cardiac surgery are crucial. Conventional renoprotective approaches have included maintaining adequate perfusion pressure, optimizing fluid management, and avoiding nephrotoxic agents [11]. However, these measures alone have not sufficiently reduced AKI incidence, prompting a search for pharmacological interventions to provide additional protection [12]. This comprehensive review examines Fenoldopam’s pharmacological properties and mechanism of action in renal protection, evaluating clinical evidence for its use in cardiac surgery, including its efficacy in preventing and treating AKI. We compare Fenoldopam to other renoprotective strategies, analyze its safety profile and cost-effectiveness, discuss current guidelines and recommendations, and identify areas for future research and potential new applications. This review aims to provide clinicians and researchers with a thorough understanding of Fenoldopam’s potential benefits and limitations, facilitating informed decision-making and highlighting areas for further investigation.

## 2. Pharmacology of Fenoldopam

**Figure 1:**
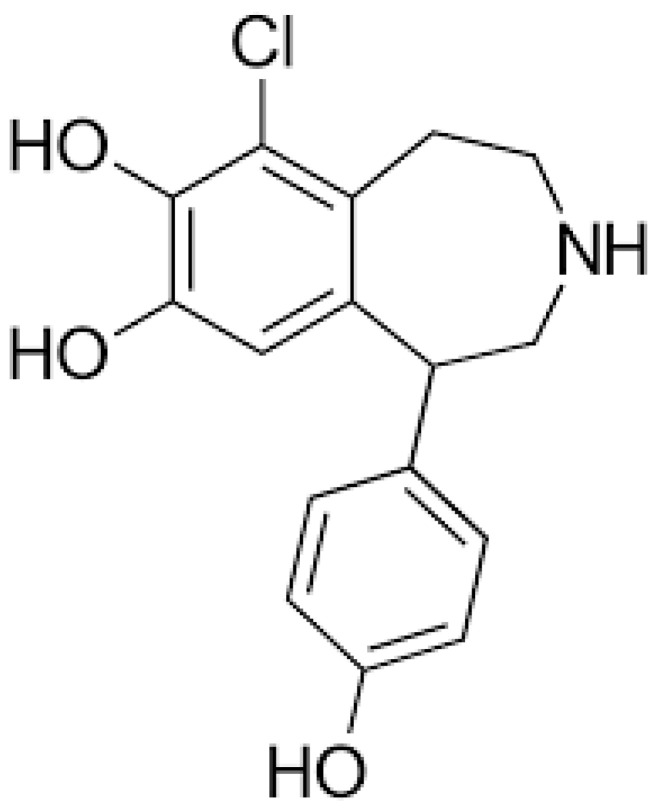
Chemical structure of Fenoldopam.

Fenoldopam is a selective dopamine-1 (D1) receptor agonist, with no significant activity at dopamine-2 (D2) or α-adrenergic receptors (Figure 1) [13]. Its primary mechanism of action involves the stimulation of D1 receptors in the renal, mesenteric, and coronary vascular beds. In the kidneys, Fenoldopam activates D1 receptors on the renal tubules and vasculature, leading to vasodilation of the afferent and efferent arterioles, as well as the arcuate and interlobular arteries [14].

This vasodilation results in increased renal blood flow, glomerular filtration rate (GFR), and sodium excretion [15]. Fenoldopam is administered intravenously [16]. It has a rapid onset of action, with hemodynamic effects observed within 5 min [17]. The drug has a short half-life of approximately 5–10 min, allowing for precise titration and quick offset of effects upon discontinuation [18]. Fenoldopam is primarily metabolized in the liver through conjugation, with no active metabolites identified. Approximately 90% of the drug is excreted in the urine within 24 h, with the remainer eliminated in feces [19]. The primary pharmacodynamic effects of Fenoldopam are related to its renal actions. At therapeutic doses, Fenoldopam increases renal blood flow by 30–40%, enhances GFR by 15–20%, and promotes natriuresis and diuresis [20]. These effects are dose-dependent and occur without significant changes in heart rate or cardiac output. In addition to its renal effects, Fenoldopam has been shown to have systemic vasodilatory properties, leading to a reduction in blood pressure [21]. This effect is particularly pronounced in hypertensive patients but is also observed to a lesser extent in normotensive individuals [22]. Fenoldopam also exhibits potential anti-inflammatory and anti-oxidative properties, which may contribute to its renoprotective effects in the setting of ischemia–reperfusion injury [23]. These properties include the suppression of pro-inflammatory cytokines and the reduction in oxidative stress markers [24]. The drug’s pharmacodynamic profile makes it particularly attractive for use in cardiac surgery, where maintaining renal perfusion and function is crucial. However, the systemic vasodilatory effects necessitate careful monitoring and potential adjustments in concurrent vasopressor therapy [6].

## 3. Renal Protection in Cardiac Surgery

The incidence of AKI in cardiac surgery varies from 8.9% to 39%, depending on the definition and patient population [1]. Understanding the intricate pathophysiology of AKI in this context is essential for developing effective renoprotective strategies. AKI development in cardiac surgery is multifactorial, involving complex interactions between patient-related risk factors and perioperative events [8]. Ischemia–reperfusion injury is a key mechanism in cardiac surgery-associated AKI. During cardiopulmonary bypass (CPB), reduced renal blood flow leads to ischemia. Reperfusion triggers a cascade of events, including reactive oxygen species (ROS) generation, inflammatory mediator activation, and mitochondrial dysfunction [25]. This oxidative stress damages cellular components, causing tubular cell injury and death. Hemodynamic instability during surgery can further compromise renal perfusion [26]. The non-pulsatile flow during CPB may also contribute to renal hypoperfusion. The systemic inflammatory response triggered by CPB is another critical factor, activating complement, coagulation cascades, and pro-inflammatory cytokines, leading to direct renal tubular injury [27]. Exposure to nephrotoxic agents in the perioperative period, including contrast media, certain antibiotics, and NSAIDs, can contribute to renal injury [28]. Hemolysis during CPB can cause tubular toxicity. Microemboli can cause renal microinfarcts [29], while hemodilution during CPB can reduce oxygen delivery to the kidneys [30]. Preexisting conditions and genetic factors may increase susceptibility to AKI. Various strategies have been employed to mitigate AKI risk, including optimizing hemodynamics [31], implementing balanced fluid management [32], minimizing exposure to harmful agents [33], and considering off-pump surgery [34]. Some studies suggest pulsatile flow during CPB may better preserve renal function [35]. Remote ischemic preconditioning has shown mixed results in conferring renoprotection [36]. Various pharmacological agents have been investigated, including N-acetylcysteine, statins, and erythropoietin, with varying success [12,37]. Despite these measures, AKI remains a significant challenge in cardiac surgery, highlighting the need for novel, comprehensive renoprotective strategies. The investigation of Fenoldopam as a potential renoprotective agent represents one such approach, targeting renal perfusion improvement and potentially offering anti-inflammatory benefits [5].

## 4. Clinical Applications of Fenoldopam in Cardiac Surgery

Fenoldopam has been investigated in various clinical contexts in cardiac surgery, aiming to preserve renal function and prevent AKI [38]. Its applications cover preoperative, intraoperative, and postoperative phases, each with unique considerations and potential benefits. Preoperatively, Cogliati et al. (2007) conducted a randomized study with 193 cardiac surgery patients [15]. Fenoldopam (0.1 μg/kg/min) or a placebo was administered for 24 h before surgery. The Fenoldopam group showed significantly lower AKI incidence (12.6% vs. 27.6%, *p* = 0.02) and shorter ICU stay, suggesting a protective effect on renal function in high-risk patients. Intraoperatively, Ranucci et al. (2010) evaluated Fenoldopam in 80 cardiac surgery patients [39]. Administered at 0.1 μg/kg/min from CPB onset until 12 h postoperatively, the Fenoldopam group demonstrated improved urine output, lower serum creatinine, and reduced AKI incidence. Postoperatively, Tumlin et al. (2009) conducted a multicenter trial with 155 cardiac surgery patients with early AKI [22]. Fenoldopam (0.1 μg/kg/min) or a placebo was given for up to 96 h. While the primary endpoint of dialysis-free survival at 21 days was not statistically significant, the Fenoldopam group showed trends towards improved renal function and reduced mortality. When using Fenoldopam, potential interactions with other renal-affecting medications must be considered. ACE inhibitors and ARBs, common in cardiovascular patients, might enhance renal vasodilation and increase hypotension risk when combined with Fenoldopam, necessitating careful blood pressure monitoring [40]. SGLT2 inhibitors, while potentially renoprotective, pose risks like euglycemic ketoacidosis perioperatively. Their interaction with Fenoldopam in cardiac surgery is not well studied, but the combination could have complementary renoprotective effects or increase risks of volume depletion and electrolyte imbalances [41]. Combining Fenoldopam with loop diuretics has shown potential synergistic effects in some studies [42], but caution is needed due to the risks of excessive diuresis and hemodynamic instability. Despite promising results, not all studies show consistent benefits. Bove et al. (2014) found no significant difference in AKI incidence between Fenoldopam and placebo groups in a large multicenter trial [43]. These conflicting findings highlight the need for further research to optimize Fenoldopam use in cardiac surgery and understand its interactions with other renal-affecting medications.

## 5. Evidence from Clinical Trials

The efficacy of Fenoldopam in preventing and treating AKI in cardiac surgery has been the subject of numerous clinical trials, yielding a mix of promising results and conflicting evidence. This section provides a comprehensive overview of the key RCTs, meta-analyses, and observational studies that have shaped our understanding of Fenoldopam’s role in this clinical context (Table 1).

Several landmark RCTs have evaluated Fenoldopam in cardiac surgery. Cogliati et al. (2007) conducted a single-center RCT involving 193 high-risk cardiac surgery patients [15]. They found that preoperative Fenoldopam infusion (0.1 μg/kg/min for 24 h before surgery) significantly reduced the incidence of AKI compared to the placebo (12.6% vs. 27.6%, *p* = 0.02). This study suggested a potential protective effect of Fenoldopam when administered preoperatively. Conversely, a large multicenter RCT by Landoni et al. (2014) in over 20 Italian hospitals found no significant difference in the incidence of AKI between Fenoldopam and placebo groups [44]. Fenoldopam was administered at 0.1 μg/kg/min from the onset of cardiopulmonary bypass for 96 h or until ICU discharge. The primary endpoint of AKI incidence was similar in both groups (Fenoldopam 20% vs. placebo 18%; *p* = 0.47). Bove et al. (2014) conducted a double-blind RCT of patients undergoing cardiac surgery with cardiopulmonary bypass [43]. They found that 20% of patients in the Fenoldopam group and 18% in the placebo group received renal replacement therapy (*p* = 0.47). Several meta-analyses have attempted to synthesize the available evidence. Landoni et al. (2007) performed a meta-analysis of 16 RCTs involving 1290 patients and found that Fenoldopam significantly reduced the risk of AKI (OR 0.43, 95% CI 0.32–0.59, *p* < 0.001) and the need for renal replacement therapy (OR 0.54, 95% CI 0.34–0.84, *p* = 0.007) [6]. A more recent meta-analysis by Gillies et al. (2015) included six RCTs specific to cardiac surgery [5]. They found that Fenoldopam was associated with a reduced incidence of AKI (RR 0.46, 95% CI 0.27–0.79) and a lower requirement for renal replacement therapy (RR 0.27, 95% CI 0.06–1.19). However, they noted significant heterogeneity among studies and emphasized the need for larger, high-quality trials. The evidence from clinical trials presents a mixed picture of Fenoldopam’s efficacy in preventing and treating AKI in cardiac surgery. While some studies have shown promising results, others have failed to demonstrate significant benefits. The heterogeneity in study designs, patient populations, dosing regimens, and definitions of AKI contribute to the difficulty in drawing definitive conclusions. Future large-scale, multicenter RCTs with standardized protocols are needed to clarify the role of Fenoldopam in this clinical setting. Additionally, identifying specific patient subgroups that may benefit most from Fenoldopam therapy remains an important area for further investigation.

## 6. Comparison with Other Renoprotective Strategies

The search for effective renoprotective strategies in cardiac surgery has led to the investigation of various pharmacological and non-pharmacological interventions. Fenoldopam’s efficacy and safety profile must be considered in the context of these alternative approaches. This section compares Fenoldopam with other commonly used or studied renoprotective strategies in cardiac surgery.

### 6.1. Dopamine

Dopamine, a precursor to Fenoldopam, was once widely used for renal protection due to its presumed ability to increase renal blood flow at low doses. However, several studies and meta-analyses have failed to demonstrate consistent benefits. Friedrich et al. (2005) conducted a meta-analysis of 61 trials involving 3359 patients and found no significant benefit of low-dose Dopamine in preventing AKI, need for dialysis, or mortality [45]. Unlike Fenoldopam, Dopamine acts on both D1 and D2 receptors, as well as α and β adrenergic receptors, leading to potentially undesirable systemic effects. Fenoldopam’s selective D1 receptor agonism may provide a more targeted renal protective effect with fewer systemic side effects compared to Dopamine [6,46].

### 6.2. Diuretics

Loop diuretics, particularly furosemide, have been widely used in an attempt to prevent or treat AKI in cardiac surgery. However, their efficacy in this context remains controversial. A meta-analysis by Ho and Power (2010) found no significant benefit of Furosemide in preventing or treating AKI in adults [47]. In contrast, Fenoldopam’s mechanism of action focuses on improving renal blood flow rather than simply increasing urine output. Some studies have suggested that the combination of Fenoldopam and Furosemide may be more effective than either agent alone in managing fluid balance and preserving renal function in critically ill patients [42].

### 6.3. N-Acetylcysteine

N-acetylcysteine (NAC) has been studied for its potential antioxidant and renoprotective effects. A meta-analysis by Adabag et al. (2008) of 10 randomized controlled trials in cardiac surgery found that NAC did not significantly reduce the incidence of postoperative AKI or mortality [48]. Some studies have explored the combination of Fenoldopam and NAC, suggesting potential synergistic effects, but further research is needed to confirm these findings [32,49].

### 6.4. Statins

Preoperative statin therapy has demonstrated potential in reducing AKI risk following cardiac surgery. A meta-analysis by Kuhn et al. (2014) revealed that preoperative statin use was associated with a lower AKI incidence (RR 0.76, 95% CI 0.67–0.87) [50]. However, a more comprehensive network meta-analysis by Chen et al. (2017) offers broader insights into the comparative efficacy of various interventions, including statins, for post-cardiac surgery AKI prevention [51]. Chen et al. analyzed 150 trials encompassing 31,322 patients and 38 pharmacological interventions. While statins showed some benefit, they were not among the top-ranked interventions. The analysis identified dexmedetomidine, corticosteroids, and aminophylline as among the most effective agents for preventing post-cardiac surgery AKI. Statins ranked in the middle range of efficacy among the studied interventions. This broader perspective suggests that although statins may provide some renoprotective effects, other pharmacological strategies might be more potent in preventing AKI in cardiac surgery contexts. The mechanism of action for statins differs from Fenoldopam, but the potential complementary effects of combining these agents warrant further investigation. Considering the complex pathophysiology of cardiac surgery-associated AKI and the potential for multi-modal renoprotective strategies, future research should explore the synergistic effects of combining statins with Fenoldopam or other renoprotective agents. This approach could lead to more comprehensive and effective strategies for preventing AKI in cardiac surgery patients.

## 7. Safety Profile and Side Effects

The use of Fenoldopam in cardiac surgery necessitates a thorough understanding of its safety profile and potential side effects. The most frequently reported side effect of Fenoldopam is hypotension, which is directly related to its vasodilatory properties [13]. In a meta-analysis by Landoni et al. (2007), the incidence of hypotension was significantly higher in patients receiving Fenoldopam compared to control groups [6]. This effect is dose-dependent and generally manageable with dose adjustment or concurrent vasopressor use. However, it underscores the importance of careful hemodynamic monitoring during Fenoldopam administration, particularly in the perioperative setting where blood pressure fluctuations can be critical. Other commonly reported side effects include tachycardia, in response to Fenoldopam-induced vasodilation [52]; headache, likely related to cerebral vasodilation [14]; flushing, for skin vasodilation [5]; nausea and vomiting [15]. These side effects are transient and resolve upon discontinuation of the drug or dose adjustment.

### Contraindications and Precautions

Fenoldopam is contraindicated in patients with known hypersensitivity to the drug or any of its components. Caution is advised in patients with glaucoma, as Fenoldopam can increase intraocular pressure; intracranial hypertension, due to cerebral vasodilation; severe aortic stenosis [20]; Fenoldopam can increase urinary potassium excretion [53]. Drug interactions are another important consideration; concomitant use of Fenoldopam with beta-blockers or other vasodilators or antihypertensive agents may result in additive hypotensive effects [43,54]. In the context of cardiac surgery, the potential for Fenoldopam to cause hypotension is of particular concern. Perioperative hypotension can compromise organ perfusion and potentially negate any renoprotective benefits. Therefore, careful titration of Fenoldopam and close hemodynamic monitoring are crucial [44] and this suggests that lower doses may offer a more favorable balance between renoprotective effects and hemodynamic stability. Long-term safety data on Fenoldopam use in cardiac surgery patients are limited, as most studies have focused on short-term perioperative use. Further research is needed to evaluate potential long-term effects or delayed complications.

## 8. Cost-Effectiveness Analysis

Evaluating the cost-effectiveness of Fenoldopam as a renoprotective strategy in cardiac surgery is crucial. While its potential benefits in reducing AKI incidence and severity are evident, the costs associated with its administration must be weighed against these benefits and compared to alternative strategies. Bove et al. (2014) incorporated economic data into their randomized controlled trial of Fenoldopam in cardiac surgery patients [43]. Although they found no significant difference in AKI incidence between Fenoldopam and placebo groups, Fenoldopam use was associated with a reduced need for renal replacement therapy (RRT), which has significant cost implications. The authors estimated potential cost savings of approximately EUR 1800 per patient, primarily due to avoided RRT costs. However, these findings should be interpreted cautiously. Fenoldopam’s cost-effectiveness may vary based on patient risk profiles, being potentially more beneficial in high-risk patients [44], local healthcare costs [4], dosing regimens, and incidence of side effects. A comprehensive cost-effectiveness analysis should consider the long-term economic impact of preventing AKI. Coca et al. (2012) showed that even mild AKI is associated with increased long-term risks of chronic kidney disease and mortality [55]. Preventing these long-term sequelae could result in substantial cost savings over time, although quantifying these benefits in the context of Fenoldopam use remains challenging. Comparing Fenoldopam’s cost-effectiveness to other renoprotective strategies is also important. For instance, remote ischemic preconditioning has shown promise in reducing AKI in cardiac surgery and may be more cost-effective due to its non-pharmacological nature [36]. The cost-effectiveness of Fenoldopam should also be compared to other pharmacological interventions such as statins or N-acetylcysteine [56]. Future cost-effectiveness analyses should incorporate data from larger, multicenter trials and consider a broader range of outcomes, including long-term renal function and quality of life measures. As personalized medicine advances, identifying specific patient subgroups in which Fenoldopam is most cost-effective will be crucial for optimizing its use in clinical practice. 

## 9. Current Guidelines and Recommendations

The use of Fenoldopam in cardiac surgery remains a topic of ongoing debate and research, resulting in varied recommendations across different guidelines and expert consensus statements. This section summarizes the current positions of major cardiovascular and nephrology societies regarding Fenoldopam use for renoprotection in cardiac surgery. The 2021 EACTS/EACTA Guidelines do not specifically recommend Fenoldopam for renoprotection [57], instead focusing on overall strategies to prevent AKI, such as maintaining adequate perfusion pressure and avoiding nephrotoxic agents. The 2012 KDIGO Clinical Practice Guideline for Acute Kidney Injury does not provide a specific recommendation for Fenoldopam use in cardiac surgery [58]. However, it suggests that Fenoldopam may be considered in high-risk patients undergoing cardiac surgery, based on low-quality evidence. The 2011 ACC/AHA Guidelines for Coronary Artery Bypass Graft Surgery do not mention Fenoldopam as a renoprotective strategy [59], likely reflecting the mixed evidence available at the time. The 2009 STS Practice Guideline Series briefly mentions Fenoldopam as a potential renoprotective agent but provides no specific recommendation for its use [60]. The 2017 ESICM consensus statement on AKI prevention mentions Fenoldopam but does not recommend its routine use for cardiac surgery-associated AKI prevention due to conflicting evidence [61]. It is important to note that the lack of strong recommendations in major guidelines does not necessarily preclude Fenoldopam’s potential benefits in specific clinical scenarios. Many guidelines acknowledge the need for individualized patient care and consideration of emerging therapies in high-risk populations [62]. Several expert consensus statements and review articles provide more nuanced recommendations, as outlined below.

A 2016 expert consensus statement suggests considering Fenoldopam in high-risk patients, particularly those with pre-existing renal dysfunction [63]. A 2018 review by Bellomo et al. mentions Fenoldopam as a potential option but emphasizes the need for further large-scale trials before widespread adoption [64]. The 2019 Canadian Society of Nephrology commentary suggests considering Fenoldopam in select high-risk cardiac surgery patients, emphasizing careful hemodynamic monitoring [65].

In conclusion, current guidelines and recommendations regarding Fenoldopam use in cardiac surgery are cautious and generally do not strongly endorse its routine use, reflecting mixed evidence from clinical trials and the need for further high-quality studies. Clinicians considering Fenoldopam as a renoprotective strategy should carefully weigh the potential benefits against risks, considering individual patient factors and institutional experiences. As new evidence emerges, future guideline updates may provide more definitive recommendations on Fenoldopam’s role in cardiac surgery-associated AKI prevention and management.

## 10. Future Directions

Various ongoing clinical trials are exploring different aspects of Fenoldopam use in cardiac surgery. The FINNAKI-FENO trial (NCT04612985), a large multicenter randomized controlled study, is assessing Fenoldopam’s efficacy in preventing AKI among high-risk cardiac surgery patients. Another trial (NCT04434482) is examining the combined effects of remote ischemic preconditioning and Fenoldopam for renoprotection, potentially offering synergistic benefits and addressing some limitations of Fenoldopam monotherapy. The KIDNEY-CARE trial (NCT04612998) is investigating a biomarker-guided approach to initiate Fenoldopam therapy in cardiac surgery patients at high risk for AKI, representing a step towards personalized medicine. Future research directions should include optimizing Fenoldopam dosing [5], exploring combination therapies with other renoprotective agents, conducting extended follow-up studies to assess long-term impacts on chronic kidney disease progression and mortality [55], investigating genetic factors [66], developing novel delivery methods [67], refining biomarker-guided therapy [63], performing cost-effectiveness analyses [4], integrating artificial intelligence, exploring non-cardiac surgery applications [68], and conducting mechanistic studies [23]. As these research avenues are pursued, it is essential to design well-powered studies that address the limitations of previous trials. The future role of Fenoldopam in cardiac surgery will likely hinge on our ability to identify suitable patients, optimize dosing regimens, and potentially combine it with other renoprotective strategies.

## 11. Conclusions

Fenoldopam, a selective dopamine-1 receptor agonist, has shown potential for renoprotection in cardiac surgery, particularly in high-risk patients, but evidence remains mixed. Safety considerations, primarily related to its vasodilatory effects, necessitate careful patient selection and close hemodynamic monitoring during administration. The role of Fenoldopam in cardiac surgery remains an area of active research and debate. While current evidence suggests potential benefits in certain patient populations, there are still significant gaps in our understanding that need to be addressed. Current guidelines do not strongly endorse routine use of Fenoldopam in cardiac surgery, reflecting the need for more definitive evidence from large-scale clinical trials. Future research directions, including biomarker-guided therapy and combination approaches, may help refine Fenoldopam’s role in preventing AKI. The decision to use Fenoldopam should be made on a case-by-case basis, considering individual patient risk factors, institutional experience, and the latest available evidence, while awaiting results from ongoing clinical trials and further research to fully elucidate its optimal use in cardiac surgery. Future research directions should focus on refining the use of Fenoldopam and exploring new avenues for renoprotection in cardiac surgery.

## Figures and Tables

**Table 1:** Summary of clinical trials on the efficacy of Fenoldopam in preventing and treating AKI in cardiac surgery.

Study	N. Patients	Dosing of Fenoldopam	Comparison	Outcomes
Cogliati et al. [15]	193 (95 Fenoldopam; 98 Placebo)	0.1 mcg/kg/min	Placebo	Fenoldopam prevented AKI in a high-risk population undergoing cardiac surgery
Ranucci et al. [39]	80 (40 Fenoldopam; 40 Placebo)	0.1 mcg/kg/min	Placebo	Fenoldopam significantly improves renal function and prevents AKI and major morbidity.
Barr et al. [32]	79 (19 Fenoldopam; 20 N-acetylcysteine; 21 Fenoldopam + N-acetylcysteine; 19 Placebo)	0.1 mcg/kg/min	N-acetylcysteine 600 mg os twice a day	Perioperative Fenoldopam and N-acetylcysteine abrogate the early postoperative decline in renal function of patients who have chronic renal insufficiency
Tumlin et al. [22]	155 (80 Fenoldopam; 75 Placebo)	0.1 mcg/kg/min	Placebo	Fenoldopam does not reduce the incidence of death or dialysis therapy in intensive care unit patients with early ATN
Bove et al. [43]	667 (338 Fenoldopam; 329 Placebo)	0.1 mcg/kg/min	Placebo	Fenoldopam did not reduce the need for renal replacement therapy or risk of 30-day mortality, but was associated with increased hypotension

AKI: acute kidney injury; ATN: acute tubular necrosis.

## Data Availability

Not applicable.

## References

[1] Hu J., Chen R., Liu S., Yu X., Zou J., Ding X. (2016). Global Incidence and Outcomes of Adult Patients with Acute Kidney Injury After Cardiac Surgery: A Systematic Review and Meta-Analysis. J. Cardiothorac. Vasc. Anesth..

[2] O’Neal J.B., Shaw A.D., Billings F.T. (2016). Acute kidney injury following cardiac surgery: Current understanding and future directions. Crit. Care.

[3] Corredor C., Thomson R., Al-Subaie N. (2016). Long-Term Consequences of Acute Kidney Injury After Cardiac Surgery: A Systematic Review and Meta-Analysis. J. Cardiothorac. Vasc. Anesth..

[4] Dasta J.F., Kane-Gill S.L., Durtschi A.J., Pathak D.S., Kellum J.A. (2008). Costs and outcomes of acute kidney injury (AKI) following cardiac surgery. Nephrol. Dial. Transplant. Off. Publ. Eur. Dial. Transpl. Assoc. Eur. Ren. Assoc..

[5] Gillies M.A., Kakar V., Parker R.J., Honoré P.M., Ostermann M. (2015). Fenoldopam to prevent acute kidney injury after major surgery-a systematic review and meta-analysis. Crit. Care.

[6] Landoni G., Biondi-Zoccai G.G., Tumlin J.A., Bove T., De Luca M., Calabrò M.G., Ranucci M., Zangrillo A. (2007). Beneficial impact of fenoldopam in critically ill patients with or at risk for acute renal failure: A meta-analysis of randomized clinical trials. Am. J. Kidney Dis. Off. J. Natl. Kidney Found..

[7] Meco M., Cirri S. (2010). The effect of various fenoldopam doses on renal perfusion in patients undergoing cardiac surgery. Ann. Thorac. Surg..

[8] Wang Y., Bellomo R. (2017). Cardiac surgery-associated acute kidney injury: Risk factors, pathophysiology and treatment. Nature reviews. Nephrology.

[9] Putaggio A., Tigano S., Caruso A., La Via L., Sanfilippo F. (2023). Red Blood Cell Transfusion Guided by Hemoglobin Only or Integrating Perfusion Markers in Patients Undergoing Cardiac Surgery: A Systematic Review and Meta-Analysis with Trial Sequential Analysis. J. Cardiothorac. Vasc. Anesth..

[10] Coca S.G., Singanamala S., Parikh C.R. (2012). Chronic kidney disease after acute kidney injury: A systematic review and meta-analysis. Kidney Int..

[11] La Via L., Vasile F., Perna F., Zawadka M. (2024). Prediction of fluid responsiveness in critical care: Current evidence and future perspective. Trends Anaesth. Crit. Care.

[12] Meersch M., Schmidt C., Zarbock A. (2017). Perioperative Acute Kidney Injury: An Under-Recognized Problem. Anesth. Analg..

[13] Murphy M.B., Murray C., Shorten G.D. (2001). Fenoldopam: A selective peripheral dopamine-receptor agonist for the treatment of severe hypertension. N. Engl. J. Med..

[14] Mathur V.S., Swan S.K., Lambrecht L.J., Anjum S., Fellmann J., McGuire D., Epstein M., Luther R.R. (1999). The effects of fenoldopam, a selective dopamine receptor agonist, on systemic and renal hemodynamics in normotensive subjects. Crit. Care Med..

[15] Cogliati A.A., Vellutini R., Nardini A., Urovi S., Hamdan M., Landoni G., Guelfi P. (2007). Fenoldopam infusion for renal protection in high-risk cardiac surgery patients: A randomized clinical study. J. Cardiothorac. Vasc. Anesth..

[16] Brogden R.N., Markham A. (1997). Fenoldopam: A review of its pharmacodynamic and pharmacokinetic properties and intravenous clinical potential in the management of hypertensive urgencies and emergencies. Drugs.

[17] Weber R.R., McCoy C.E., Ziemniak J.A., Frederickson E.D., Goldberg L.I., Murphy M.B. (1988). Pharmacokinetic and pharmacodynamic properties of intravenous fenoldopam, a dopamine1-receptor agonist, in hypertensive patients. Br. J. Clin. Pharmacol..

[18] Allison N.L., Dubb J.W., Ziemniak J.A., Alexander F., Stote R.M. (1987). The effect of fenoldopam, a dopaminergic agonist, on renal hemodynamics. Clin. Pharmacol. Ther..

[19] Chertow G.M., Sayegh M.H., Allgren R.L., Lazarus J.M. (1996). Is the administration of dopamine associated with adverse or favorable outcomes in acute renal failure? Auriculin Anaritide Acute Renal Failure Study Group. Am. J. Med..

[20] Halpenny M., Rushe C., Breen P., Cunningham A.J., Boucher-Hayes D., Shorten G.D. (2002). The effects of fenoldopam on renal function in patients undergoing elective aortic surgery. Eur. J. Anaesthesiol..

[21] Shusterman N.H., Elliott W.J., White W.B. (1993). Fenoldopam, but not nitroprusside, improves renal function in severely hypertensive patients with impaired renal function. Am. J. Med..

[22] Tumlin J.A., Finkel K.W., Murray P.T., Samuels J., Cotsonis G., Shaw A.D. (2005). Fenoldopam mesylate in early acute tubular necrosis: A randomized, double-blind, placebo-controlled clinical trial. Am. J. Kidney Dis. Off. J. Natl. Kidney Found..

[23] Aravindan N., Natarajan M., Shaw A.D. (2006). Fenoldopam inhibits nuclear translocation of nuclear factor kappa B in a rat model of surgical ischemic acute renal failure. J. Cardiothorac. Vasc. Anesth..

[24] Lavalle S., Masiello E., Iannella G., Magliulo G., Pace A., Lechien J.R., Calvo-Henriquez C., Cocuzza S., Parisi F.M., Favier V. (2024). Unraveling the Complexities of Oxidative Stress and Inflammation Biomarkers in Obstructive Sleep Apnea Syndrome: A Comprehensive Review. Life.

[25] Bastin A.J., Ostermann M., Slack A.J., Diller G.P., Finney S.J., Evans T.W. (2013). Acute kidney injury after cardiac surgery according to Risk/Injury/Failure/Loss/End-stage, Acute Kidney Injury Network, and Kidney Disease: Improving Global Outcomes classifications. J. Crit. Care.

[26] Tigano S., Caruso A., Liotta C., LaVia L., Vargas M., Romagnoli S., Landoni G., Sanfilippo F. (2024). Exposure to severe hyperoxemia worsens survival and neurological outcome in patients supported by veno-arterial extracorporeal membrane oxygenation: A meta-analysis. Resuscitation.

[27] Rosner M.H., Okusa M.D. (2006). Acute kidney injury associated with cardiac surgery. Clin. J. Am. Soc. Nephrol. CJASN.

[28] Medalion B., Cohen H., Assali A., Vaknin Assa H., Farkash A., Snir E., Sharoni E., Biderman P., Milo G., Battler A. (2010). The effect of cardiac angiography timing, contrast media dose, and preoperative renal function on acute renal failure after coronary artery bypass grafting. J. Thorac. Cardiovasc. Surg..

[29] Sreeram G.M., Grocott H.P., White W.D., Newman M.F., Stafford-Smith M. (2004). Transcranial Doppler emboli count predicts rise in creatinine after coronary artery bypass graft surgery. J. Cardiothorac. Vasc. Anesth..

[30] Karkouti K., Beattie W.S., Wijeysundera D.N., Rao V., Chan C., Dattilo K.M., Djaiani G., Ivanov J., Karski J., David T.E. (2005). Hemodilution during cardiopulmonary bypass is an independent risk factor for acute renal failure in adult cardiac surgery. J. Thorac. Cardiovasc. Surg..

[31] Azau A., Markowicz P., Corbeau J.J., Cottineau C., Moreau X., Baufreton C., Beydon L. (2014). Increasing mean arterial pressure during cardiac surgery does not reduce the rate of postoperative acute kidney injury. Perfusion.

[32] Barr L.F., Kolodner K. (2008). N-acetylcysteine and fenoldopam protect the renal function of patients with chronic renal insufficiency undergoing cardiac surgery. Crit. Care Med..

[33] Mariscalco G., Lorusso R., Dominici C., Renzulli A., Sala A. (2011). Acute kidney injury: A relevant complication after cardiac surgery. Ann. Thorac. Surg..

[34] Garg A.X., Devereaux P.J., Yusuf S., Cuerden M.S., Parikh C.R., Coca S.G., Walsh M., Novick R., Cook R.J., Jain A.R. (2014). Kidney function after off-pump or on-pump coronary artery bypass graft surgery: A randomized clinical trial. JAMA.

[35] Mahmoud A.B., Burhani M.S., Hannef A.A., Jamjoom A.A., Al-Githmi I.S., Baslaim G.M. (2005). Effect of modified ultrafiltration on pulmonary function after cardiopulmonary bypass. Chest.

[36] Zarbock A., Schmidt C., Van Aken H., Wempe C., Martens S., Zahn P.K., Wolf B., Goebel U., Schwer C.I., Rosenberger P. (2015). Effect of remote ischemic preconditioning on kidney injury among high-risk patients undergoing cardiac surgery: A randomized clinical trial. JAMA.

[37] Chen J.J., Lee T.H., Kuo G., Huang Y.T., Chen P.R., Chen S.W., Yang H.Y., Hsu H.H., Hsiao C.C., Yang C.H. (2022). Strategies for post-cardiac surgery acute kidney injury prevention: A network meta-analysis of randomized controlled trials. Front. Cardiovasc. Med..

[38] Sun H., Xie Q., Peng Z. (2019). Does Fenoldopam Protect Kidney in Cardiac Surgery? A Systemic Review and Meta-Analysis With Trial Sequential Analysis. Shock.

[39] Ranucci M., De Benedetti D., Bianchini C., Castelvecchio S., Ballotta A., Frigiola A., Menicanti L. (2010). Effects of fenoldopam infusion in complex cardiac surgical operations: A prospective, randomized, double-blind, placebo-controlled study. Minerva Anestesiol..

[40] Mancia G., Fagard R., Narkiewicz K., Redon J., Zanchetti A., Böhm M., Christiaens T., Cifkova R., De Backer G., Dominiczak A. (2013). 2013 ESH/ESC guidelines for the management of arterial hypertension: The Task Force for the Management of Arterial Hypertension of the European Society of Hypertension (ESH) and of the European Society of Cardiology (ESC). Eur. Heart J..

[41] Nadkarni G.N., Ferrandino R., Chang A., Surapaneni A., Chauhan K., Poojary P., Saha A., Ferket B., Grams M.E., Coca S.G. (2017). Acute Kidney Injury in Patients on SGLT2 Inhibitors: A Propensity-Matched Analysis. Diabetes Care.

[42] Pannu N., Nadim M.K. (2008). An overview of drug-induced acute kidney injury. Crit. Care Med..

[43] Bove T., Zangrillo A., Guarracino F., Alvaro G., Persi B., Maglioni E., Galdieri N., Comis M., Caramelli F., Pasero D.C. (2014). Effect of fenoldopam on use of renal replacement therapy among patients with acute kidney injury after cardiac surgery: A randomized clinical trial. JAMA.

[44] Landoni G., Bove T., Pasero D., Comis M., Orando S., Pinelli F., Guarracino F., Corcione A., Galdieri N., Zucchetti M. (2010). Fenoldopam to prevent renal replacement therapy after cardiac surgery. Design of the FENO-HSR study. HSR Proc. Intensive Care Cardiovasc. Anesth..

[45] Friedrich J.O., Adhikari N., Herridge M.S., Beyene J. (2005). Meta-analysis: Low-dose dopamine increases urine output but does not prevent renal dysfunction or death. Ann. Intern. Med..

[46] Sorbello M., Morello G., Paratore A., Cutuli M., Mistretta G., Belluoccio A.A., Veroux M., Veroux P., Macarone M., Gagliano M. (2007). Fenoldopam vs dopamine as a nephroprotective strategy during living donor kidney transplantation: Preliminary data. Transplant. Proc..

[47] Ho K.M., Power B.M. (2010). Benefits and risks of furosemide in acute kidney injury. Anaesthesia.

[48] Adabag A.S., Ishani A., Bloomfield H.E., Ngo A.K., Wilt T.J. (2009). Efficacy of N-acetylcysteine in preventing renal injury after heart surgery: A systematic review of randomized trials. Eur. Heart J..

[49] Sorbello M., Morello G., Parrinello L., Molino C., Rinzivillo D., Pappalardo R., Cutuli M., Corona D., Veroux P., Veroux M. (2010). Effect of N-acetyl-cysteine (NAC) added to fenoldopam or dopamine on end-tidal carbon dioxide and mean arterial pressure at time of renal artery declamping during cadaveric kidney transplantation. Transplant. Proc..

[50] Kuhn E.W., Liakopoulos O.J., Stange S., Deppe A.C., Slottosch I., Choi Y.H., Wahlers T. (2014). Preoperative statin therapy in cardiac surgery: A meta-analysis of 90,000 patients. Eur. J. Cardio-Thorac. Surg. Off. J. Eur. Assoc. Cardio Thorac. Surg..

[51] Chen X., Huang T., Cao X., Xu G. (2018). Comparative Efficacy of Drugs for Preventing Acute Kidney Injury after Cardiac Surgery: A Network Meta-Analysis. Am. J. Cardiovasc. Drugs.

[52] Tumlin J.A., Wang A., Murray P.T., Mathur V.S. (2002). Fenoldopam mesylate blocks reductions in renal plasma flow after radiocontrast dye infusion: A pilot trial in the prevention of contrast nephropathy. Am. Heart J..

[53] Caimmi P.P., Pagani L., Micalizzi E., Fiume C., Guani S., Bernardi M., Parodi F., Cordero G., Fregonara M., Kapetanakis E. (2003). Fenoldopam for renal protection in patients undergoing cardiopulmonary bypass. J. Cardiothorac. Vasc. Anesth..

[54] Allgren R.L., Marbury T.C., Rahman S.N., Weisberg L.S., Fenves A.Z., Lafayette R.A., Sweet R.M., Genter F.C., Kurnik B.R., Conger J.D. (1997). Anaritide in acute tubular necrosis. Auriculin Anaritide Acute Renal Failure Study Group. N. Engl. J. Med..

[55] Coca S.G., Yusuf B., Shlipak M.G., Garg A.X., Parikh C.R. (2009). Long-term risk of mortality and other adverse outcomes after acute kidney injury: A systematic review and meta-analysis. Am. J. Kidney Dis. Off. J. Natl. Kidney Found..

[56] Billings F.T.t., Hendricks P.A., Schildcrout J.S., Shi Y., Petracek M.R., Byrne J.G., Brown N.J. (2016). High-Dose Perioperative Atorvastatin and Acute Kidney Injury Following Cardiac Surgery: A Randomized Clinical Trial. JAMA.

[57] Wahba A., Milojevic M., Boer C., De Somer F., Gudbjartsson T., van den Goor J., Jones T.J., Lomivorotov V., Merkle F., Ranucci M. (2020). 2019 EACTS/EACTA/EBCP guidelines on cardiopulmonary bypass in adult cardiac surgery. Eur. J. Cardio-Thorac. Surg. Off. J. Eur. Assoc. Cardio Thorac. Surg..

[58] Khwaja A. (2012). KDIGO clinical practice guidelines for acute kidney injury. Nephron. Clin. Pract..

[59] Hillis L.D., Smith P.K., Anderson J.L., Bittl J.A., Bridges C.R., Byrne J.G., Cigarroa J.E., Disesa V.J., Hiratzka L.F., Hutter A.M. (2011). 2011 ACCF/AHA Guideline for Coronary Artery Bypass Graft Surgery: A report of the American College of Cardiology Foundation/American Heart Association Task Force on Practice Guidelines. Circulation.

[60] Lazar H.L., McDonnell M., Chipkin S.R., Furnary A.P., Engelman R.M., Sadhu A.R., Bridges C.R., Haan C.K., Svedjeholm R., Taegtmeyer H. (2009). The Society of Thoracic Surgeons practice guideline series: Blood glucose management during adult cardiac surgery. Ann. Thorac. Surg..

[61] Joannidis M., Druml W., Forni L.G., Groeneveld A.B.J., Honore P.M., Hoste E., Ostermann M., Oudemans-van Straaten H.M., Schetz M. (2017). Prevention of acute kidney injury and protection of renal function in the intensive care unit: Update 2017: Expert opinion of the Working Group on Prevention, AKI section, European Society of Intensive Care Medicine. Intensive Care Med..

[62] La Via L., Sangiorgio G., Stefani S., Marino A., Nunnari G., Cocuzza S., La Mantia I., Cacopardo B., Stracquadanio S., Spampinato S. (2024). The Global Burden of Sepsis and Septic Shock. Epidemiologia.

[63] Meersch M., Schmidt C., Hoffmeier A., Van Aken H., Wempe C., Gerss J., Zarbock A. (2017). Prevention of cardiac surgery-associated AKI by implementing the KDIGO guidelines in high risk patients identified by biomarkers: The PrevAKI randomized controlled trial. Nat. Rev. Nephrol..

[64] Bellomo R., Ronco C., Mehta R.L., Asfar P., Boisramé-Helms J., Darmon M., Diehl J.L., Duranteau J., Hoste E.A.J., Olivier J.B. (2017). Acute kidney injury in the ICU: From injury to recovery: Reports from the 5th Paris International Conference. Intensive Care Med..

[65] James M., Bouchard J., Ho J., Klarenbach S., LaFrance J.P., Rigatto C., Wald R., Zappitelli M., Pannu N. (2013). Canadian Society of Nephrology commentary on the 2012 KDIGO clinical practice guideline for acute kidney injury. Am. J. Kidney Dis. Off. J. Natl. Kidney Found..

[66] Tang W.H., Vagelos R.H., Yee Y.G., Fowler M.B. (2004). Impact of angiotensin-converting enzyme gene polymorphism on neurohormonal responses to high- versus low-dose enalapril in advanced heart failure. Am. Heart J..

[67] Hou J., Pan Y., Zhu D., Fan Y., Feng G., Wei Y., Wang H., Qin K., Zhao T., Yang Q. (2019). Targeted delivery of nitric oxide via a ‘bump-and-hole’-based enzyme-prodrug pair. Nat. Chem. Biol..

[68] Goren O., Matot I. (2015). Perioperative acute kidney injury. Br. J. Anaesth..

